# Antibody induction in mice by liposome-displayed recombinant enterotoxigenic *Escherichia coli* (ETEC) colonization antigens

**DOI:** 10.1016/j.bj.2023.03.001

**Published:** 2023-03-15

**Authors:** Shiqi Zhou, Karl O.A. Yu, Moustafa T. Mabrouk, Dushyant Jahagirdar, Wei-Chiao Huang, Julio A. Guerra, Xuedan He, Joaquin Ortega, Steven T. Poole, Eric R. Hall, Oscar G. Gomez-Duarte, Milton Maciel, Jonathan F. Lovell

**Affiliations:** a Department of Biomedical Engineering, State University of New York at Buffalo, Buffalo, NY, USA; b Division of Pediatrics Infectious Diseases, Department of Pediatrics, University at Buffalo, Buffalo, NY, USA; c Department of Anatomy and Cell Biology, McGill University, Montreal, Canada; d Naval Medical Research Center, Silver Spring, MD, USA; e Henry M. Jackson Foundation for the Advancement of Military Medicine, Bethesda, MD, USA; f Department of Microbiology and Immunology, Uniformed Services University Health System, Bethesda, MD, USA

**Keywords:** Diarrhea, ETEC, Colonizing factor, Liposomes, Vaccine, Adjuvant

## Abstract

**Background:**

Enterotoxigenic *Escherichia coli* (ETEC) strains cause infectious diarrhea and colonize host intestine epithelia via surface-expressed colonization factors. Colonization factor antigen I (CFA/I), a prevalent ETEC colonization factor, is a vaccine target since antibodies directed to this fimbria can block ETEC adherence and prevent diarrhea.

**Methods:**

Two recombinant antigens derived from CFA/I were investigated with a vaccine adjuvant system that displays soluble antigens on the surface of immunogenic liposomes. The first antigen, CfaEB, is a chimeric fusion protein comprising the minor (CfaE) and major (CfaB) subunits of CFA/I. The second, CfaEad, is the adhesin domain of CfaE.

**Results:**

Owing to their His-tag, recombinant CfaEB and CfaEad, spontaneously bound upon admixture with nanoliposomes containing cobalt-porphyrin phospholipid (CoPoP), as well as a synthetic monophosphoryl lipid A (PHAD) adjuvant. Intramuscular immunization of mice with sub-microgram doses CfaEB or CfaEad admixed with CoPoP/PHAD liposomes elicited serum IgG and intestinal IgA antibodies. The smaller CfaEad antigen benefitted more from liposome display. Serum and intestine antibodies from mice immunized with liposome-displayed CfaEB or CfaEad recognized native CFA/I fimbria as evidenced by immunofluorescence and hemagglutination inhibition assays using the CFA/I-expressing H10407 ETEC strain.

**Conclusion:**

These data show that colonization factor-derived recombinant ETEC antigens exhibit immunogenicity when delivered in immunogenic particle-based formulations.

Enterotoxigenic *Escherichia coli* (ETEC) is a major cause of acute infectious diarrhea in children under the age of five in low and middle-income countries, as well as travelers and military personnel visiting those countries. Currently, there is no licensed vaccine against ETEC and the World Health Organization considers the development of an ETEC vaccine a priority in the prevention of ETEC-mediated diarrheal disease [1,2]. ETEC is a rod-shaped Gram-negative bacterium that initiates infection through the colonization of the host intestine via surface expressed colonization factors (CFs). Colonization factor antigen I (CFA/I) is the most broadly expressed ETEC CF and a member of the class 5 fimbrial family, which includes the related coli surface antigens CS1, CS2, CS4, CS14, CS17, CS19, and PCFO71 [3,4]. The structure of CFA/I fimbriae comprises over 1000 major subunits of CfaB, and a minor subunit of the tip adhesin CfaE [5]. CfaE itself is a two-domain protein with an N-terminal adhesin domain (ad) and a C-terminal pilin domain (pd). The major subunit CfaB interacts with CfaE through donor protein strand complementation with the pd [6]. Immunization with recombinant CfaE can elicit functional antibodies (Abs) that neutralize bacterial binding by targeting the adhesin domain, CfaEad, which contains the putative binding site [7]. Vaccine approaches using CFA/I subunits [[8], [9], [10]] have included CfaB- or CfaE-derived multi-epitope fusion antigens [11,12], the minor subunit CfaE [13,14], and a fusion of CfaE and CfaB (CfaEB) [15].

Oral immunization is generally the standard paradigm for administration of ETEC vaccines, as it is likely to elicit mucosal Ab responses within the gut. In a clinical phase 1/2 trial, the inactivated ETEC vaccine ETVAX, which includes *E. coli* that overexpress the CFs CFA/I, CS3, CS5, and CS6 along with the B-subunit of the cholera toxin, proved to be safe and tolerable in children with oral delivery of two doses [16] Moreover, the administration of ETVAX with the double mutant of the heat-labile toxin (dmLT) enhanced the magnitude of the immune response [17]. Of note, while native ETEC heat-labile toxin (LT) exhibits toxicity that is incompatible with its use as antigen and/or adjuvant, dmLT is considered a safe and effective mucosal and parenteral adjuvant [[18], [19], [20]]. However, oral vaccination of infants in developing countries tends to yield poor immunity, which could be related to poor nutritional supply, frequent exposure to enteric pathogens or other factors [21]. Oral vaccines also need to overcome the significant barriers of bile acid and proteolytic enzymes in the gut. High doses are required which may increase costs of vaccine development or even potentially increase the risk of inducing tolerance effects [22]. Parenteral immunization against ETEC has been proposed as an alternative strategy and has been shown to induce mucosal immune responses [13,23]. Of note, a recent study in mice demonstrated that the intramuscular route can induce systemic and mucosal immune responses using a CFA/I subunit vaccine [24]. Other parenteral delivery routes such as transcutaneous or intradermal immunization for ETEC subunit vaccines have also been reported [25,26].

The use of soluble, recombinant ETEC subunits is appealing for rational vaccine development. However, purified proteins tend to be poorly immunogenic, necessitating the use of adjuvants to boost responses. Liposomes are biodegradable nanovesicles that have been developed as clinical stage vaccine adjuvants including GSK's AS01 adjuvant [27,28]. As our group reported previously, cobalt porphyrin-phospholipid (CoPoP) can be incorporated into liposomes to enable the spontaneous binding of his-tagged peptides and proteins [[29], [30], [31], [32], [33]]. CoPoP liposomes are co-formulated with the synthetic monophosphoryl lipid A (MPLA) adjuvant Phosphorylated HexaAcyl Disaccharide 3D6A (PHAD-3D6A, abbreviated herein simply as PHAD) and can enhance immunogenicity of vaccines [24]. We hypothesized that binding between CoPoP liposomes and his-tagged antigens could increase their delivery to antigen presenting cells (APCs), which will lead to strong immune responses. As a comparison, the widely used adjuvant Alum was included in this study. Alum has been applied with various antigens as a low cost and safe adjuvant [34,35]. The goal of the current study was to explore the adjuvanticity of CoPoP/PHAD liposomes containing ETEC CF subunit-derived recombinant antigens, CfaEB or CfaEad by evaluating the systemic and mucosal immune responses elicited by intramuscular immunization of mice with low doses of antigen in either soluble or liposome particle format.

## Results

### Recombinant his-tagged ETEC antigens bind to CoPoP/PHAD liposomes

**Fig. 1:**
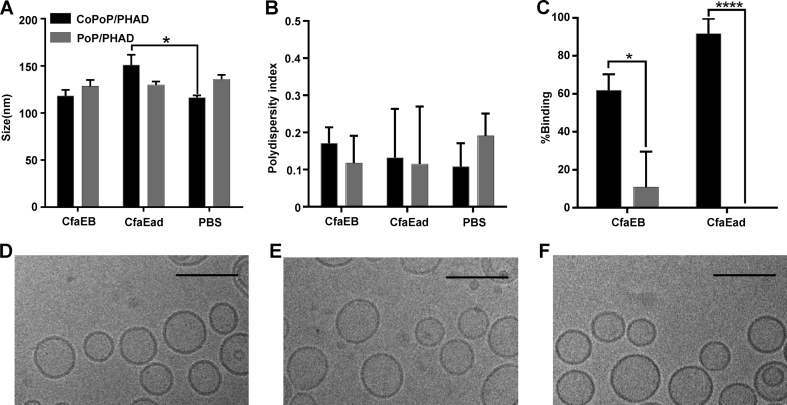
**Recombinant colonization factor antigens spontaneously bind to CoPoP/PHAD liposomes.** CfaEB or CfaEad was admixed with liposomes for 3 h at room temperature and the resulting liposome **(A)** Size **(B)** Polydispersity index, and **(C)** Percentage of CfaEB and CfaEad binding to liposomes were assessed. Values show mean ± std. dev. for triplicate measurements. Cryo-electron micrographs of CoPoP/PHAD liposomes decorated with **(D)** CfaEB **(E)** CfaEad or **(F)** without antigen. Scale bar represents 100 nm. Statistical comparison for C was performed by unpaired t test, ∗*p* < 0.05, ∗∗∗∗*p* < 0.0001.

In contrast to PoP/PHAD liposomes, CoPoP/PHAD liposomes contain chelated cobalt in the bilayer. This intrabilayer cobalt results in the biostable binding of recombinant his-tagged proteins [36]. Binding of his-tagged, recombinant CfaEB and CfaEad to CoPoP/PHAD liposomes was assessed. The size of the liposomes with or without incubation with proteins remained in the range of 100–150 nm during the process [Fig. 1A]. Polydispersity values were less than 0.2, reflecting a reasonably monodisperse particle size population that was generally unaffected by CfaEB or CfaEad binding [Fig. 1B]. Approximately 60% of CfaEB and 90% of CfaEad bound to CoPoP/PHAD liposomes as assessed by a microcentrifugal filtration assay [Fig. 1C]. In contrast, less than 10% protein binding was observed to the analogous, cobalt deficient, PoP/PHAD liposomes. When assessed by cryo-electron microscopy, no individual proteins were visualized, which is not unexpected given their small size. Liposomes bound with CfaEB [Fig. 1D] or CfaEad [Fig. 1E] appeared highly spherical with no obvious difference with naked CoPoP/PHAD liposomes in size and shape [Fig. 1F].

**Fig. 2:**
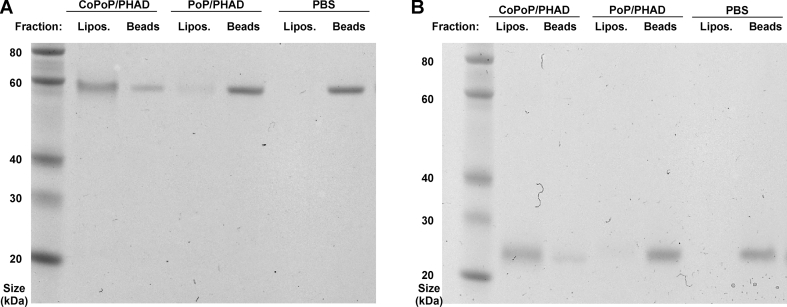
**Recombinant ETEC antigens stably bind CoPoP/PHAD liposomes.** Liposomes were admixed with **(A)** CfaEB or **(B)** CfaEad for 3 h, then combined with Ni-NTA magnetic beads for 30 min at room temperature. Beads were separated from supernatant and SDS-PAGE was used to determine whether the proteins were in the liposome (“Lipos.“) fraction (bound to liposomes) or the Ni-NTA Beads fraction (unbound to liposomes).

Binding of the antigens with liposomes was confirmed by a Ni-NTA magnetic bead competition assay. In this assay, the proteins in particle or soluble form are incubated with Ni-NTA beads, which sequester any unbound, his-tagged proteins. After admixture of both CfaEB [Fig. 2A] and CfaEad [Fig. 2B] with CoPoP/PHAD liposomes, the proteins remained in the supernatant which contains the liposome-bound proteins, whereas little protein was found in the Ni-NTA bead pellet, which binds to soluble protein. Ni-NTA beads captured CfaEB and CfaEad alone in phosphate buffered saline (PBS) or after admixture with PoP/PHAD liposomes lacking cobalt. Based on band densitometry analysis, CoPoP/PHAD liposomes bound 79% of CfaEB and 84% CfaEad, whereas PoP/PHAD liposomes bound only 12% and 5% of the same proteins, respectively.

### Liposome-displayed proteins improve delivery to antigen-presenting cells

**Fig. 3:**
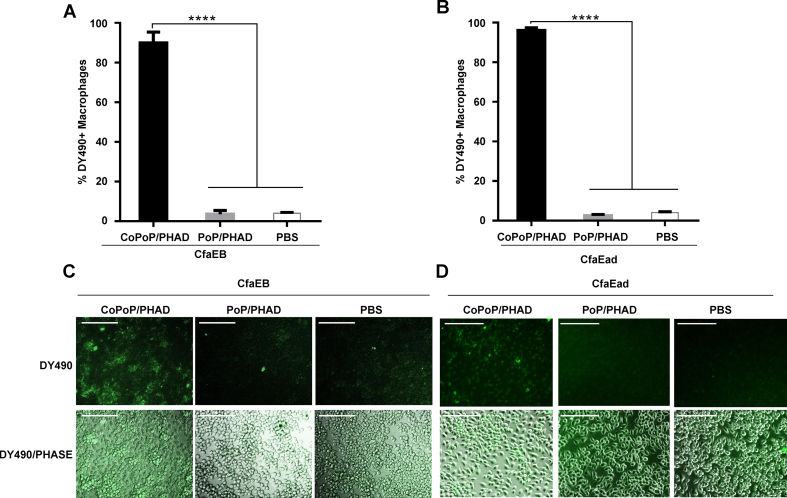
**Enhanced *in vitro* uptake of CfaEB and CfaEad in macrophages with liposome-display.** RAW264.7 macrophages were incubated with CfaEB-490 or CfaEad-490 admixed with CoPoP/PHAD or PoP/PHAD liposomes and uptake was assessed. **(A)** Percentage of CfaEB-490^+^ and **(B)** CfaEad-490^+^ macrophages assessed by flow cytometry. Fluorescence microscopy of macrophages after incubation with pre-incubated (**C**) CfaEB-490^+^- or (**D**) CfaEad-490^+^- with indicated liposomes. Scale bars represent 100 μm. Representative images from three independent experiments are shown. Graphs show mean ± std. dev. for three independent experiments. Statistical comparisons were performed by one-way ANOVA followed by Tukey's multiple comparisons test, ∗∗∗∗*p* < 0.0001.

We previously found that one of the mechanisms by which the CoPoP system modulate the immune response is by enhancing antigen delivery to APCs *in vitro* and *in vivo* [37]. Hence, we assessed *in vitro* macrophage uptake of the two recombinant ETEC antigens. CfaEB and CfaEad proteins were labeled with the fluorescent dye DY-490-NHS-Ester to generate the fluorescent CfaEB-490 and CfaEad-490 antigens, respectively. The fluorescent proteins were admixed with PoP/PHAD or CoPoP/PHAD liposomes and incubated with a murine macrophage cell line. Based on flow cytometry, less than 4% of macrophages took up free CfaEB-490 and less than 6% of macrophages took up CfaEB-490 when admixed with PoP/PHAD liposomes lacking cobalt. In contrast, approximately 90% of macrophages showed substantial CfaEB-490 uptake when the protein was admixed with CoPoP/PHAD liposomes (*p* < 0.0001, Fig. 3A). For CfaEad-490, CoPoP/PHAD liposomes also significantly increased CfaEad-490 uptake from 2% to over 95% of macrophages (*p* < 0.0001, Fig. 3B). Macrophages were visualized with fluorescence microscopy to confirm the uptake of CfaEB-490 [Fig. 3C] and CfaEad-490 [Fig. 3D]. Altogether, the results indicate that admixture with liposomes containing cobalt enhances uptake of his-tagged proteins by macrophages *in vitro*.

### CoPoP/PHAD liposomes improve the immunogenicity of CFA/I subunit proteins

**Fig. 4:**
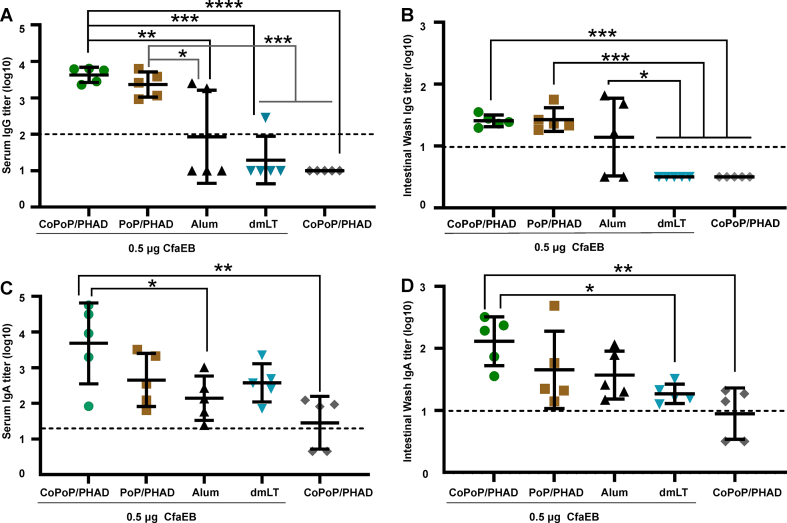
**Enhanced vaccine-induced antibody response following CfaEB admixture with CoPoP/PHAD liposomes.** Outbred mice were vaccinated IM on day 0 and 21 with 0.5 μg of CfaEB admixed with Alum dmLT adjuvants or liposomes (CoPoP/PHAD or PoP/PHAD). Sera and intestinal washes (IW) were collected on day 42 for analysis. **(A)** Serum anti-CfaEB IgG titers **(B)** IW anti-CfaEB IgG titers (**C)** Serum anti-CfaEB IgA titers (**D)** IW anti-CfaEB IgA titers. The dashed line in each figure indicates the starting dilution factor of each assay. Data points represent individual values for each mouse, and mean and standard deviation are also indicated. Statistical analysis was performed by one-way ANOVA followed by Tukey's multiple comparisons test using log_10_-transformed values for n = 5 mice/group, ∗*p* < 0.05, ∗∗*p* < 0.01, ∗∗∗*p* < 0.001 and ∗∗∗∗*p* < 0.0001.

To assess the immunogenicity of different adjuvants, CfaEB or CfaEad antigens were admixed with CoPoP/PHAD, PoP/PHAD liposomes, Alum, or dmLT adjuvants separately. A group of mice vaccinated with protein-free CoPoP/PHAD liposomes as a control. Mice were immunized intramuscularly with 0.5 μg antigen per injection on day 0 and 21, and sera and intestinal washes (IW) were collected on day 42. Antigen-specific IgG and IgA titers in sera and IW were measured by ELISA. Serum anti-CfaEB IgG levels were significantly elevated in the CfaEB-CoPoP/PHAD group compared to the CfaEB-Alum, CfaEB-dmLT and CoPoP/PHAD-only groups (*p* < 0.05, *p* < 0.01, and *p* < 0.001 respectively; Fig. 4A). For the dmLT groups, high levels of anti-dmLT IgG were observed in sera (Figure S1 in the Supporting Information). IW anti-CfaEB IgG titers in the CfaEB-CoPoP/PHAD and CfaEB-PoP/PHAD groups were significantly higher than CfaEB-dmLT and CoPoP/PHAD-only groups (*p* < 0.001; Fig. 4B). The average serum anti-CfaEB IgA titer in the CfaEB-CoPoP/PHAD group was significantly higher than the CfaEB-Alum and CoPoP/PHAD-only groups (*p* < 0.05 and *p* < 0.01, respectively; Fig. 4C). Although anti-CfaEB IgA titers were low overall in IW, the highest titers were detected in the CfaEB-CoPoP/PHAD group, which were significantly elevated in comparison to CfaEB-dmLT and CoPoP/PHAD-only group (*p* < 0.05 and *p* < 0.01 respectively; Fig. 4D).

**Fig. 5:**
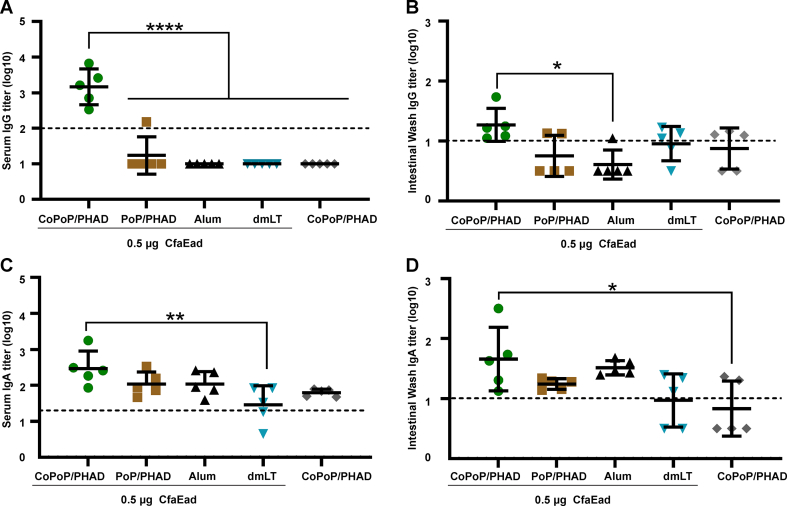
**Enhanced vaccine-induced antibody response following CfaEad admixture with CoPoP/PHAD liposomes.** Outbred mice were vaccinated on day 0 and 21 with 0.5 μg CfaEad admixed with the indicated adjuvants. Serum and IW were collected on day 42 for Ab assessment. **(A)** Serum anti-CfaEad IgG titers **(B)** IW anti-CfaEad IgG titers **(C)** Serum anti-CfaEad IgA titers **(D)** IW anti-CfaEad IgA titers. The dashed line in each figure indicates the starting dilution factor of each assay. Values below the dash line were extrapolated. Mean and standard deviation are indicated. Statistical analysis performed with Log_10_-transformed values (n = 5 mice/group) with statistical comparisons performed by one-way ANOVA followed by Tukey's multiple comparisons test, ∗*p* < 0.05, ∗∗*p* < 0.01 and ∗∗∗∗*p* < 0.0001.

Serum anti-CfaEad IgG levels were significantly elevated in CfaEad-CoPoP/PHAD group compared to all other groups (*p* < 0.0001; Fig. 5A). Regardless of the overall low titers observed in the IW, CfaEad-CoPoP/PHAD groups showed the highest average anti-CfaEad IgG titer, which was significantly higher than the CfaEad-Alum group (*p* < 0.05, Fig. 5B). The CfaEad-CoPoP/PHAD group showed significantly higher intestinal wash IgG titer than the CfaEad-PoP/PHAD group by unpaired t-test analysis (*p* = 0.0304, Fig. 5B). The average anti-CfaEad IgA serum titer in the CfaEad-CoPoP/PHAD group was significantly higher than observed in the CfaEad-CoPoP/PHAD and CfaEad-dmLT groups (*p* < 0.01; Fig. 5C). In the IW, the IgA titer was the highest in the CfaEad-CoPoP/PHAD group, but this was only significantly higher than the protein-free CoPoP/PHAD control group (*p* < 0.05; Fig. 5D).

### Intramuscular immunization with CfaEB- or CfaEad-CoPoP/PHAD elicits functional antibodies

**Fig. 6:**
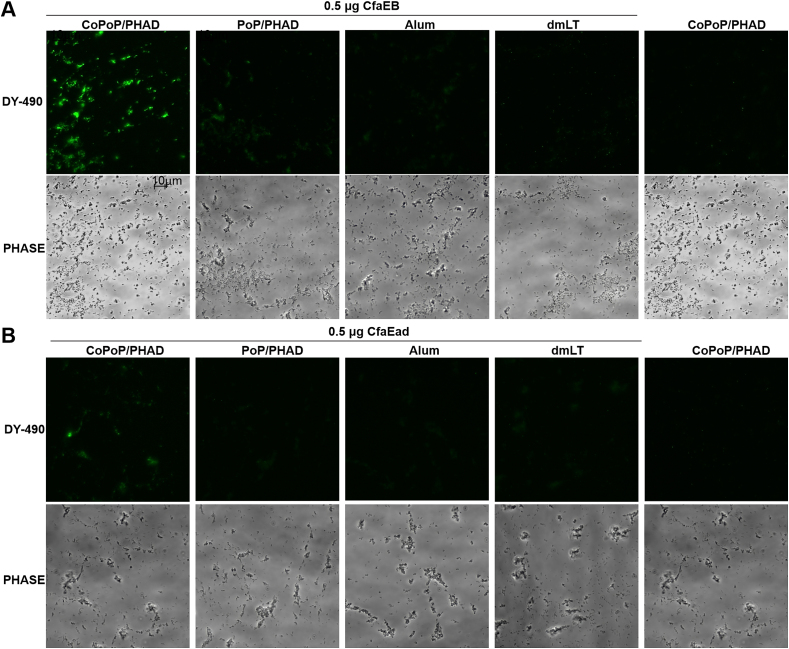
**Binding of antibodies in post-immune serum to CFA/I** ^**+**^**ETEC bacteria visualized by an indirect fluorescence assay.** Fixed CFA/I^+^ ETEC bacteria (strain H10407) were incubated with pooled post-immune serum, followed by incubation with Alexa 488-labeled secondary Ab. H10407 bacteria under fluorescence microscopy with the use of **(A)** anti-CfaEB or **(B)** anti-CfaEad serum Abs. Scale bars indicate 10 μm. Representative images from two independent assays with pooled sera (from n = 5 mice) are shown.

An indirect fluorescent assay (IFA) was used to investigate the binding profile of anti-CfaEB and anti-CfaEad Abs to CFA/I^+^ ETEC (strain H10407). The bacteria were fixed and then incubated with sera from vaccinated mice. Bacteria incubated with the post-immune sera from the CfaEB-CoPoP/PHAD (Fig. 6A) and CfaEad-CoPoP/PHAD [Fig. 6B] groups displayed strong fluorescence. This reflects that Abs recognized the native antigens. The higher Ab levels induced by the CoPoP/PHAD groups compared to controls is due to the transformation of his-tagged antigen into particles via CoPoP liposome binding, which better deliver the antigens to APCs. Nearly negligible fluorescence bacteria were visible in the antigen-PoP/PHAD, antigen-Alum, antigen-dmLT and CoPoP/PHAD-only groups post-immune sera samples. In comparison to the sera from the CfaEad group, the observed bacterial fluorescence intensity was increased with serum from the CfaEB group. This is likely due to the contribution of the anti-fimbrial (anti-CfaB) Abs, which would not be present in the anti-CfaEad sera, binding to the CFA/I structure on the bacterial surface.

**Fig. 7:**
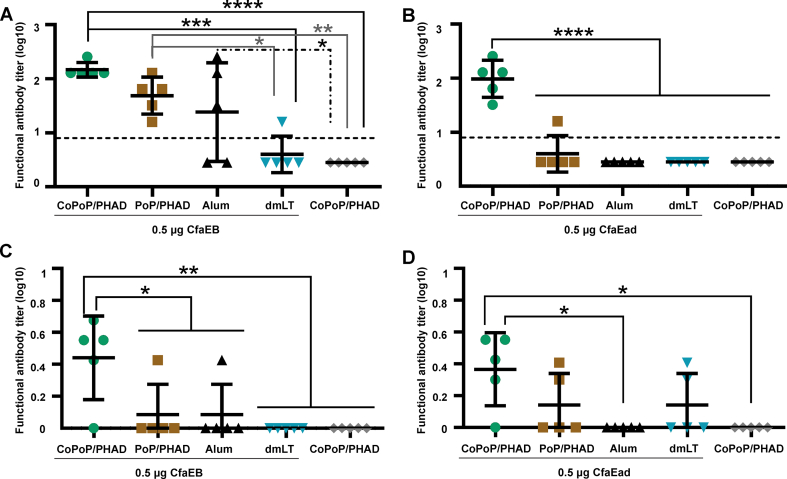
**Immunization with CfaEB- and CfaEad admixed with CoPoP/PHAD liposomes elicits neutralizing antibodies in serum and intestinal washes**. Mice were vaccinated on days 0 and 21 through the IM route, and serum and IW were collected on day 42. The activity of anti-CFA/I neutralizing Abs was assessed by a hemagglutination inhibition (HAI) assay with bovine red blood cells. **(A, C)** HAI results in serum and IW, respectively, from groups immunized with CfaEB plus different adjuvants **(B, D)** HAI results in serum and IW, respectively, from groups immunized with CfaEad plus different adjuvants. Horizontal solid lines represent the mean of each group (n = 5/group). Dash line in figure A and B indicate the starting dilution factor of each assay. Statistical comparisons were performed by one-way ANOVA followed by Tukey's multiple comparisons test, ∗*p* < 0.05, ∗∗*p* < 0.01, ∗∗∗*p* < 0.001 and ∗∗∗∗*p* < 0.0001.

The agglutination of bovine erythrocytes by CFA/I^+^ ETEC (strain H10407) bacteria in the presence of mannose can be prevented *in vitro* by pre-incubating the bacteria with functional neutralizing Abs [38,39]. Both serum and IW Abs elicited by IM immunization with CfaEB or CfaEad complexed with CoPoP/PHAD inhibited the hemagglutination activity caused by the bacteria. Serum from the CfaEB-CoPoP/PHAD group contained significantly higher functional Ab titers compared to serum from the CfaEB-dmLT and CoPoP/PHAD-only groups (*p* < 0.001 and *p* < 0.0001 respectively; Fig. 7A); A significant difference was detected between CfaEB-CoPoP/PHAD and CfaEB-PoP/PHAD groups by unpaired t-test (*p* = 0.0193, Fig. 7A). Serum samples of CfaEB-PoP/PHAD group contained higher serum functional Ab titer than CfaEB-dmLT and CoPoP/PHAD only group (*p* < 0.05 and *p* < 0.01 respectively; Fig. 7A). The inhibition of hemagglutination was more pronounced for serum samples of mice vaccinated with CfaEad-CoPoP/PHAD than of mice vaccinated with CfaEad-PoP/PHAD, -Alum or -dmLT adjuvants (*p* < 0.0001 to all comparisons; Fig. 7B). IW samples only showed neutralization activity with very low dilutions. IW neutralizing Ab levels in CfaEB-CoPoP/PHAD group were significantly higher compared to PoP/PHAD, Alum and dmLT, CoPoP/PHAD only group (*p* < 0.05, *p* < 0.05, *p* < 0.01 and *p* < 0.01 respectively; Fig. 7C). The use of CfaEad-CoPoP/PHAD liposomes led to significantly higher titer of IW neutralizing Abs than CfaEad-Alum and CoPoP/PHAD only group (*p* < 0.05 and *p* < 0.05 respectively; Fig. 7D), though there were not statistically different from CfaEad-PoP/PHAD or CfaEad-dmLT groups. Based on the IFA and hemagglutination inhibition, post-immune serum and IW from mice immunized with CfaEB and CfaEad complexed with CoPoP/PAHD recognized and neutralized CFA/I^+^ ETEC bacteria (strain H10407). Moreover, at the antigen dose used, the results suggest that CoPoP/PHAD adjuvant system outperformed Alum and dmLT in improving both systemic and mucosal functional Ab induction.

### Variation in vaccine dosage and adjuvants

To demonstrate the reproducibility of these findings and to assess strategies to overcome low antibody levels in IW, immunization with increased antigen and adjuvant doses was carried out. Mice were immunized with the original vaccination schedule and CfaEad dose of 0.5 μg, as well as with higher doses of 1.5 μg and 5 μg. As the ratio between antigen and liposome was fixed, the PHAD was correspondingly increased. However, CfaEad-specific serum IgG titer (Fig. S2A in the Supporting Information), IgA titer (Fig. S2B), and the IW IgG titer (Fig. S2C), and IgA titer (Fig. S2D) did not increase their magnitude despite the higher dose. The vaccine dose of 0.5 μg was sufficient to achieve a maximal antigen-specific antibody response with this particular vaccine formulation. To test whether QS-21 adjuvant could increase CfaEad-specific antibody titer, we compared PoP/PHAD liposomes, CoPoP/PHAD liposomes, CoPoP/PHAD/QS-21 liposomes and CoPoP/PHAD/QS-21 liposomes by immunizing mice with 5 μg CfaEad antigen admixed with these liposomes (Fig. S3A in the Supporting Information). A significant difference was detected between the CoPoP/PHAD and CoPoP/PHAD/QS-21 group serum IgG titer by unpaired t test (*p* < 0.01), with QS-21 enabling higher antibody levels to be induced. The CoPoP/PHAD and CoPoP/PHAD/QS-21 group IW IgG also showed significant difference (*p* < 0.05; Fig. S3C). Both serum (Fig. S3B) and IW (Fig. S3D) CfaEad-specific IgA titer was increased with the CoPoP/PHAD/QS-21 liposomes, but it was not statistically significant. Liposome formulations that omitted cobalt but otherwise contained the same PHAD and QS-21 adjuvants were not effective and antibody levels remained nearly at baseline levels.

## Discussion

In this study, we explored the use of CoPoP/PHAD and PoP/PHAD liposomes for displaying two different soluble recombinant CFA/I-derived antigens, namely, CfaEB and CfaEad. Their immunogenicity was assessed following intramuscular administration to mice. Both proteins, CfaEad and CfaEB, effectively bound to CoPoP/PHAD liposomes upon simple admixture, as judged by filtration and Ni-NTA competition assays. However, since the filtration assay revealed that the majority, but not all the antigen, bound to the liposomes, future work should focus on defining the best conditions to ensure the complete conversion of the soluble antigens into particles by either optimizing admixture conditions (e.g. ratio liposome/antigen), or by addition of a purification step. It seems reasonable to speculate that those conditions will be different for each antigen, which can be affected by size as well as conformational factors that affect the accessibility of the His tag.

Both CfaEad and CfaEB, exhibited enhanced *in vitro* uptake by macrophages when complexed with CoPoP/PHAD liposomes, which could partially account for any observed enhanced immunogenicity where it was observed. Some differences between the two CFA/I-derived antigens are noteworthy. The CfaEB fusion protein contains both the minor CfaE (including CfaEad) and the major CfaB subunit of CFA/I. Antibodies targeting the different components of CfaEB, especially anti-CfaB Abs, would be able to bind multiple site on the CFA/I fimbriae. In contrast, Abs induced by CfaEad could only target the CfaE adhesin domain, which offers an explanation as to why antibodies induced by CfaEB-CoPoP/PHAD reacted to a greater extent with the CFA/I^+^ ETEC bacteria in the IFA study. Furthermore, as judged by ELISA results, CfaEB tended to be more immunogenic when administered with cobalt-free PoP/PHAD liposomes, Alum or dmLT adjuvants, while CfaEad was virtually non-immunogenic with all adjuvants except CoPoP/PHAD, at 0.5 μg/dose. The smaller size of CfaEad may render the protein a hapten-like antigen, which shows greater benefit from particle-based immunization. CfaEB appeared to exhibit slightly better uptake in macrophages *in vitro* compared to CfaEad.

At sub-microgram dosing, CfaEB and CfaEad antigens induced greater levels of serum and IW functional neutralizing antibodies when complexed with CoPoP/PHAD compared to other adjuvants. Anti-CfaEB Abs levels appear lower than seen in a recent report also using IM immunizations plus an MPLA-containing adjuvant, GLA-SE [24]. Although a direct comparison of the results is not possible given the difference of the antigen dose (0.5 vs 6.3 μg/dose) and possible methodological variations, our results indicate that similar immune responses can be elicited with less antigen when CoPoP/PHAD is employed. Functional neutralizing antibodies detected by the hemagglutination Inhibition assay indicate the humoral response targeted the bacteria fimbriae to prevent the agglutination of red blood cells caused by ETEC bacteria [[40], [41], [42], [43]]. Functional antibody responses were also observed in IW, which lends support to the concept that the parenteral route can potentially induce a mucosal Ab response [23,44].

While this study demonstrated that liposome-based adjuvant systems could be a viable platform for the parenteral delivery of ETEC vaccine antigens, several areas of future work should be noted. The safety and stability of the liposome formulations were not assessed. While we did not notice any change in the animals after immunization, pre-clinical safety might be better assessed with a non-human primate (NHP) model. In the future, it would also be important to evaluate the immunogenicity and efficacy of a CfaEB- or CfaEad-CoPoP/PHAD vaccine in a NHP model of ETEC, for example the *Aotus nancymaae* model [45].

Given the small amount of antigen that is necessary based on the CoPoP/PHAD liposomal system and the ability of CoPoP/PHAD liposomes to produce multiplexed particles that induce increased Abs response [[46], [47], [48]], combining multiple ETEC antigens in a single vaccine could be another important future direction. Given ETEC strain diversity, a multivalent vaccine would be a logical next step and other ETEC antigens could include coli surface antigen 6 subunit antigen CssBA, for example [49,50]. Other future work could involve assessment of the distribution of induced Ab subclasses; cellular cytokine response to immunization; and investigation of other parenteral vaccination route, such as subcutaneous or intradermal injection.

One of the major limitations of this study is that the IW antibody titer levels were low. An attempt to increase them by using higher CfaEad dosing did not lead to increased serum IgG nor IW IgA level (Fig. S2 in the Supporting Information). Inclusion of a second lipid-phase adjuvant in addition to PHAD (QS-21) resulted in significantly increased IgG antibody levels in serum and intestine wash (Fig. S3 in the Supporting Information). However, IgA levels did not significantly increase. Overall, further work to improve intestinal antibody levels should be explored. Possible directions include changing the route of delivery. Recently, antigens displayed on CoPoP liposomes were shown to induce antigen-specific mucosal IgA responses in the lungs, following intranasal immunization of mice, which could be another avenue of exploration in the future [51,52]. Alternatively, other vaccine adjuvants might more effectively lead to better intestinal IgA responses.

In this study, we demonstrated that the display of recombinant CFA/I antigens (CfaEad and CfaEB) on the surface of CoPoP/PHAD liposomes can elicit systemic and mucosal responses against ETEC using low antigen doses. Moreover, since the liposome system takes advantage of His-tagged antigens which are simple to purify and characterize, this could be used to facilitate the rapid advancement of new vaccine candidates into proof-of-concept clinical trials. We note that the CoPoP liposome system has been recently progressed through Phase 2 clinical trials for a COVID-19 vaccine (ClincalTrials.gov identifier NCT04783311).

## Conclusion

In this study, CoPoP/PHAD liposomes were shown to provide an effective route for developing parenterally-delivered subunit-based ETEC vaccines in mice. Particle formulations were readily formed upon admixing recombinant his-tagged protein antigens and CoPoP/PHAD liposomes. This resulted in enhanced delivery of CfaEB or CfaEad to macrophages *in vitro*, which might have contributed to the enhancement of antigen-specific IgG and IgA antibodies in sera and IW following immunization. The induced Abs in response to sub-microgram immunization could bind to ETEC strain H10407 as judged by IFA and hemagglutination inhibition. CF antigen subunit vaccines delivered in particle format by admixture with CoPoP/PHAD liposome appears to be a worthwhile strategy for further research studies.

## Materials and methods

### Liposome preparation

CoPoP and PoP were produced as recently described [53]. Other lipids used for liposomes production were 1,2-dipalmitoyl-sn-glycero-3-phosphocholine (DPPC, Corden # LP-R4-057), cholesterol (PhytoChol, Wilshire Technologies), synthetic monophosphoryl lipid A 3D6A Phosphorylated HexaAcyl Disaccharide (3D6A-PHAD, abbreviated simply as PHAD, Avanti Cat # 699855 P), 1,2-dipalmitoyl-sn-glycero-3-phosphocholine (DOPC, Corden # LP-R4-076), and QS-21 (DesertKing). CoPoP/PHAD and PoP/PHAD liposomes were produced by ethanol injection and nitrogen-pressurized lipid extrusion [54]. Briefly, lipids were dissolved in 1 mL pre-heated 50 °C ethanol for 10 min and then diluted with pre-heated PBS (50 °C) to 5 mL and incubated at 50 °C for 10 min. The 1 mg/mL CoPoP and liposomes were filtered through 0.45 μm filter and then extruded through 200,100 and 80 nm membrane filters stack for 15 times. The liposome extruder (Northern Lipids) were nitrogen pressurized and used at 50 °C with a pressure of near 200 PSI followed by dialyzing in PBS at 4 °C twice to remove ethanol. Liposomes were passed through a 0.2 μm sterile filter and adjusted to 320 μg/mL CoPoP before stored at 4 °C. Liposome formulations were based on the mass ratio of DPPC: CHOL: CoPoP (or PoP): PHAD as 4:2:0.4:1. Experiments shown in Fig. S2 and Fig. S3 in the Supporting Information used a modified formulation based on DOPC: CHOL: CoPoP (or PoP): PHAD:QS-21 (20:5:1:0.4:0.4). Liposomes that included QS-21 were prepared by admixing QS-21 with PoP/PHAD or CoPoP/PHAD liposomes at 4 °C overnight.

### Protein and bacteria preparation

Recombinant proteins CfaEB and CfaEad with 6*X* his-tags were produced in bacteria as previously described [55,56]. Colonization factor antigen I (CFA/I) expressing strain Enterotoxigenic *Escherichia coli* H10407 and non-expressing strain DH5α were kept on dry ice once taken out from −80 °C storage. In sterile conditions, frozen bacteria were collected with a sterilized wooden stick the and dipped into 1 mL LB broth with gently mix by pipette. 200 μL bacteria solution was spread on CFA agar plates followed by culturing in the incubator at 37 °C overnight.

### Animal and biospecimen collection

Mice were purchased from Envigo and maintained at the Comparative Medicine and Laboratory Animal Facilities (CM-LAF) of the University at Buffalo, State University of New York (IACUC Protocol# BME05044Y). CD1 (ICR) mice (6–7 weeks old, female) were randomly separated to five animals per group and vaccinated at day 0 and 21 through intramuscular route with 0.5 μg proteins with indicated adjuvants. At day 42, all mice were anesthetized with isoflurane for serum collection and sacrificed by cervical dislocation under anesthesia for intestinal wash collection. Mouse serum were separated by centrifugation at 1200*g* for 15 min under room temperature. Clear serum samples stored at 4 °C or −80 °C for long term storage. The small intestine was removed and was flushed with 2 mL sterilized cold PBS containing 1% protease inhibitor (ThermoFisher Scientific, Halt Protease Inhibitor Cocktail, Cat # 78,425). Intestinal wash was filtered through a 0.45 μm membrane filter (Nanosep MF Centrifugal device). Clear intestinal wash samples were stored at −80 °C until use.

### Cell line and cell culture

RAW264.7 macrophage cells were purchased from the ATCC and cultured in Dulbecco's modified Eagle's medium with 10% fetal bovine serum (FBS) and 1% 100 U/mL penicillin and 100 μg/mL streptomycin. Cells were cultured at 37 °C with 5% CO_2_.

### Vaccine preparation and mice immunization

Proteins (80 μg/mL in PBS) were admixed with CoPoP/PHAD liposomes or PoP/PHAD liposomes (320 μg/mL CoPoP or PoP) at room temperature for 3 h. Alum (Invivogen Cat # vac-alu-250) was diluted to a final concentration of 1.5 mg/mL in HEPES buffer (pH 7.4) after mixing with antigen. The dmLT protein adjuvant (BEI Resources NR-51682, R192G/L211A mutations) was resuspend with 1 mL of 0.22 μm sterile filtered water. For each mouse in dmLT group, 0.5 μg dmLT was admixed with antigen before vaccination per mouse per injection. All vaccines were diluted to 0.5 μg CF antigen per 50 μL by PBS before immunizing mice with intramuscular injection into the left hind quadriceps pm day 0 and 21. CoPoP/PHAD only group mice received same dose of CoPoP/PHAD liposomes as protein-CoPoP/PHAD group without any protein. Mice received 2 μg CoPoP and 2 μg PHAD in CoPoP/PHAD liposomes, 2 μg of PoP and 2 μg PHAD in PoP/PHAD liposomes, or 0.5 μg adjuvant dmLT in a final volume of 50 μL. CoPoP/PHAD and PoP/PHAD liposomes were co-incubated with CfaEB and CfaEad at 4:1 PHAD: protein mass ratio for 3 h under room temperature before injection. Alum and dmLT were admixed with proteins separately before injection. Endotoxin levels in CfaEB and CfaEad were assessed using the Pierce Chromogenic Endotoxin Quant Kit (Thermo Scientific, Cat # A39552) according to manufacturer instructions. At the injection concentration, both proteins had less than 0.1 EU/mL of endotoxin.

### Protein and liposome binding assay

Antigen (80 μg/mL) was admixed with CoPoP/PHAD or PoP/PHAD liposomes (320 μg/mL CoPoP or PoP) for 3 h at room temperature. Samples were diluted to 0.5 μg protein per 50 μL (equivalent to the vaccination dose). Size and polydispersity index of liposome were measured by NanoBrook 90Plus PALS instrument by diluting 2 μL liposomes with or without proteins into 1 mL PBS. For determining binding, samples were centrifuged at 12,000 rcf for 1 h at 4 °C. Supernatants were collected for protein concentration measurement. Binding percentage was calculated according to the protein left in the supernatant determined by BCA kit (ThermoFisher Scientific, Cat # 23,225) with the formula: Percent of binding = [1-(CoPoP/PHAD or PoP/PHAD supernatant/Protein only supernatant)] × 100%.

### Ni-NTA challenge assay

Co-incubated protein-liposomes (2 μg proteins) were mixed with 5 μL PBS washed Ni-NTA magnetic beads (ThermoFisher Scientific, Cat # 88,831) for 30 min at room temperature. Supernatant and beads were separated by magnetic separator (ThermoFisher # 12321D). Beads were re-suspended with 20 μL PBS. Denaturing PAGE loading dye was mixed with supernatant or beads samples separately and heated at 100 °C for 10 min. Samples were loaded onto a Novex 4–12% Bis-Tris acrylamide gel (Invitrogen # NP0321) and subjected to PAGE followed by visualizing band with Coomassie staining. Quantification analysis of Ni-NTA challenge results were processed by Image Lab Software (Bio-Rad). The percentage of bound protein was calculated as the (band volume of bound protein)/(volume of bound protein + volume of unbound protein) × 100%. The percentage of unbound protein was calculated in a similar manner as the (band volume of unbound protein)/(volume of bound protein + volume of Unbound protein) × 100%.

### Cryo-EM microscopy

A volume of 25 μL of CfaEB or CfaEad proteins (80 μg/mL) were mixed with 25 μL of CoPoP/PHAD liposomes (320 μg/mL CoPoP) and incubated at room temperature for 3 h followed by adding 150 μL PBS. For CoPoP/PHAD only liposome, 25 μL of CoPoP/PHAD liposomes were added into 175 μL of PBS buffer. Sample vitrification was performed using a Vitrobot Mark IV (Thermo Fisher Scientific). Holey carbon grids (C-Flat 2/2-3Cu-T) were washed with chloroform for 2 h prior to sample vitrification. Grids were treated with negative glow discharge in air at 5 mA for 15 s right before the sample was applied. For all samples, a volume of 3.6 μL of the liposome sample was applied to a holey carbon grid and manually blotted using the Vitrobot blotting paper (Standard Vitrobot Filter Paper, Ø55/20 mm, Grade 595). Then a volume of 3.6 μL of the same sample was applied for a second time to the same holey carbon grid and the grid was blotted once in the Vitrobot for 3 s using a blot force +1, before plunging it into liquid ethane. The Vitrobot was set at 25 °C and 100% relative humidity. Grids were loaded into a Tecnai F20 electron microscope operated at 200 kV using a Gatan 626 single tilt side entry cryo-holder at FEMR-McGill. The data acquisition was performed on TVIPS XF416 CMOS camera using the Serial-EM software. All images were collected at a magnification of 62,000*x* which produced images with a calibrated pixel size of 1.761 Å. Images were collected with a total dose of ∼50 e^−/^Å^2^ using a defocus ranging from −1.75 to −2.75 μm.

### *In vitro* macrophage uptake

CfaEB and CfaEad were separately labeled with DY-490-NHS-Ester (DY-490, Dyomics) for macrophage uptake studies. 100 μL of proteins (1 mg/mL) were dialyzed in 100 mM NaHCO_3_ buffer (pH 9) overnight at 4 °C. Dialyzed proteins were mixed with 20 μL DY-490 for 1 h under room temperature followed by dialyzing the sample in PBS overnight at 4 °C. Protein concentration measured by BCA kit (ThermoFisher Scientific, Cat # 23,225) after the labeling. Labeled proteins were covered with foil and stored at 4 °C. 5 × 10^5^/well RAW264.7 macrophage cells were seeded in sterilized 24-well plate overnight to reach 70–80% confluence. Culture medium was replaced with incomplete DMEM (without additives) for 3 h incubation. Macrophages were co-incubated with 1 μg CfaEB-490 or CfaEad-490 alone or 1 μg CfaEB-490 or CfaEad-490 with 4 μg CoPoP/PHAD or PoP/PHAD liposomes for 3 h. Cells were washed with PBS for 3 times. Macrophages in 500 μL PBS were imaged by fluorescence microscopy then detached by cell scraper and gently re-suspended with pipetting for flow cytometry.

## ELISA

CfaEB and CfaEad proteins were diluted with coating buffer (100 mM Na/CO_3_ buffer, pH 9.6) to 1 μg/mL. One hundred μL were added into each well of 96-well plates for 2 h at 37 °C. ELISA plates were washed with PBS with 0.05% Tween 20 (PBST) for 3 times then were blocked with 2% BSA in PBST for 1 h at 37 °C. 100 μL series diluted mouse serum or intestinal wash samples (in 1% BSA) were added into each well for 1 h at 37 °C followed by 3 PBST wash. Goat anti mouse IgG or IgA secondary Abs (100 μL, 1:1000 diluted in 1% BSA) were added and co-incubated for 30min at 37 °C. ELISA plates were washed with PBST for 6 times. TMB (tetramethylbenzidine, 100 μL) solution were added into each well and incubated at room temperature for 15min for IgG signal development or at 37 °C for 45 min for IgA signal development. A volume of 100 μL 1 M HCl were applied to stop the reaction. Absorbance of samples were measure at 450 nm. The antigen specific IgG and IgA titer were defined as the highest dilution of serum or IW that achieved an OD450 of 0.5 of IgG titers or 0.1 for IgA titers.

### Indirect fluorescence antibody (IFA) assay

ETEC bacteria H10407 and DH5α were cultured overnight on Colonization Factor Antigen (CFA) agar plates. Bacteria were harvested and re-suspended in 1 mL PBS. Bacteria suspension (200 μL per slide) was air dried and fixed by passing slides through a flame quickly 4 times. Slides were block with 2% BSA in PBS for 30 min at room temperature followed by washing slides with PBS. Serum (pooled serum sample from the same vaccinated groups) was diluted to 1:1500 in 1% BSA before apply 200 μL serum on each smear of the bacteria for 1 h at room temperature followed by 3 PBS wash. Alexa Fluor 488 goat anti mouse secondary Ab (ThermoFisher Cat # A28175) was diluted to 1:2000 in 1%BSA. Secondary Abs (200 μL) was applied to each slide for 30 min under room temperature and covered with aluminum foil. Slides were wash with PBS for 3 times before applying the mounting medium and cover slide. Seal slides with nail polish and stored at 4 °C before visualization.

### Hemagglutination inhibition (HAI) assay

The hemagglutination inhibition (HAI) assay followed previous studies [26]. Washed Bovine Red Blood Cells, 10% suspension (Lampire Biological Laboratoris, Donor # 11,865), was stored at 4 °C for use up to 2 weeks after received. CFA/I expressing ETEC bacteria H10407 and CFA/I non-expressing ETEC bacteria DH5α were cultured overnight on Colonization Factor Antigen (CFA) agar plates. Bacterial were harvested and resuspended in 1 mL 1*x* PBS with 0.5% d-Mannose (PBSM). Bacteria was dilute with PBSM until 1:100 suspension reached OD650 at 0.2. Bacterial were kept on ice. The 10% bovine erythrocyte was washed with cold PBS for 3 times and centrifuged at 1000 rpm for 5 min for each wash. Bovine erythrocyte was diluted to 1.5% with cold PBSM. Serum and intestinal wash samples were serial diluted with PBSM. A volume of 25 μL bacteria suspension was co-incubated with 25 μL serial diluted test samples in 96-well microtest U bottom plate (Falcon Cat # 353,077) with the use of plate shaker at 1000 rpm (Fisherbrand, Microplate Shaker, cat # 88,861,023) for 30 min under room temperature. A volume of 25 μL 1.5% erythrocyte were add to each well with the use of plate shaker at 1000 rpm for 30 min in 4 °C environments. The hemagglutination inhibition (HAI) titer was defined as the highest dilution of serum or intestinal wash specimen where inhibition of the agglutination effect of *E. coli* H10407 on the bovine erythrocytes was observed.

### Statistical analysis

Data were analyzed by GraphPad Prism 9 as indicated in each caption. ∗, ∗∗, ∗∗∗, and ∗∗∗∗ indicate *p* < 0.05, 0.01, 0.001, and 0.0001, respectively. All values were reported as means ± standard deviation (S.D.). Data in Fig. 4, Fig. 5, Fig. 7 were analyzed with logarithmic transformed values.

## Declaration of competing interest

The authors declare the following financial interests/personal relationships which may be considered as potential competing interests: Wei-Chiao Huang and Jonathan Lovell are named co-inventors on one or more University at Buffalo patent applications describing CoPoP technology and hold equity in POP Biotechnologies, a university startup company licensing the technology.
