# Supplementary material for: Antibody induction in mice by liposome-displayed recombinant enterotoxigenic Escherichia coli (ETEC) colonization antigens
Source: Biomed J. 2023 Mar 15;46(6):100588. doi: 10.1016/j.bj.2023.03.001 (PMC10711177; doi:10.1016/j.bj.2023.03.001)
Supplement: Multimedia component 1 [file mmc1.docx]

# Supporting Information: Antibody Induction in Mice by Liposome-Displayed Recombinant Enterotoxigenic Escherichia coli (ETEC) Colonization Antigens

Shiqi Zhou, Karl O. A. Yu, Moustafa T. Mabrouk, Dushyant Jahagirdar, Wei-Chiao Huang, Julio A. Guerra, Xuedan He, Joaquin Ortega, Steven T. Poole, Eric R. Hall, Oscar G. Gomez-Duarte, Milton Maciel Jr.^*^, Jonathan F. Lovell^*^


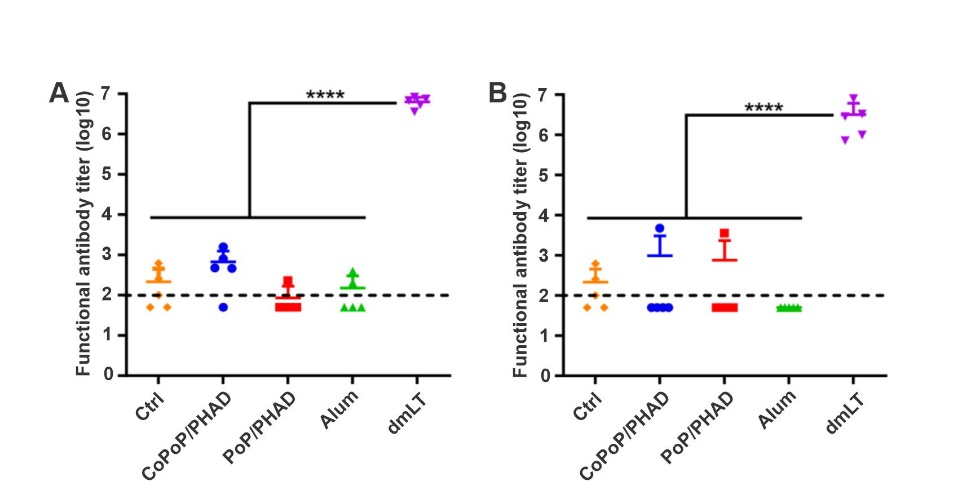


**Figure S1. Antibody response induced by dmLT.** Anti-dmLT IgG antibody titer of **A)** CfaEB and **B)** CfaEad serum samples. Statistical comparisons were performed by one-way ANOVA followed by Tukey's multiple comparisons test using log10-transformed values for n=5 mice/group, ****p<0.0001.


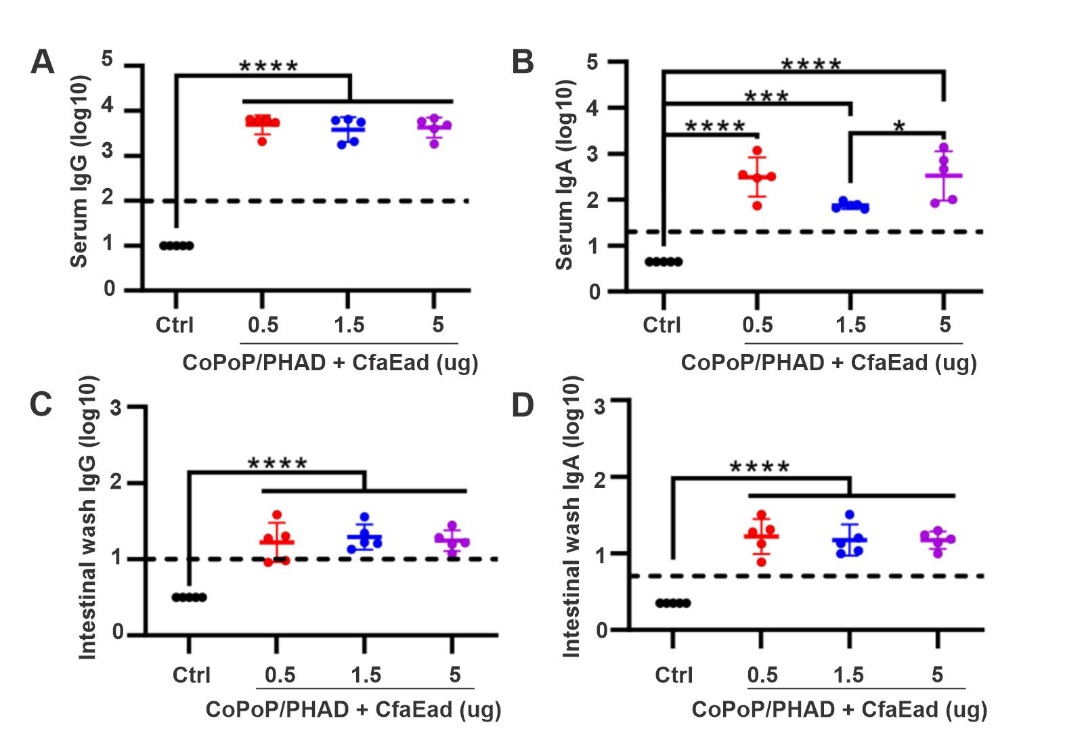


**Figure S2. CfaEad-specific IgG and IgA titer levels induced by different CfaEad doses.** ICR mice were immunized with indicated dose of CfaEad with CP liposomes on day 0 and 21. The CoPoP dose was 4-fold the antigen dose and the PHAD dose was 1.6-fold the antigen dose. Serum and intestinal wash samples were collected on day 42. **A)** Serum IgG titer; **B)** Serum IgA titer; **C)** Intestinal wash IgG titer; **D)** Intestinal wash IgA titer. Mean and standard deviation are indicated. Statistical analysis was performed with Log_10_-transformed values (n=5 mice/group) with comparisons performed by one-way ANOVA and Tukey's multiple comparisons test, *p<0.05, ***p<0.001, and ****p<0.0001.


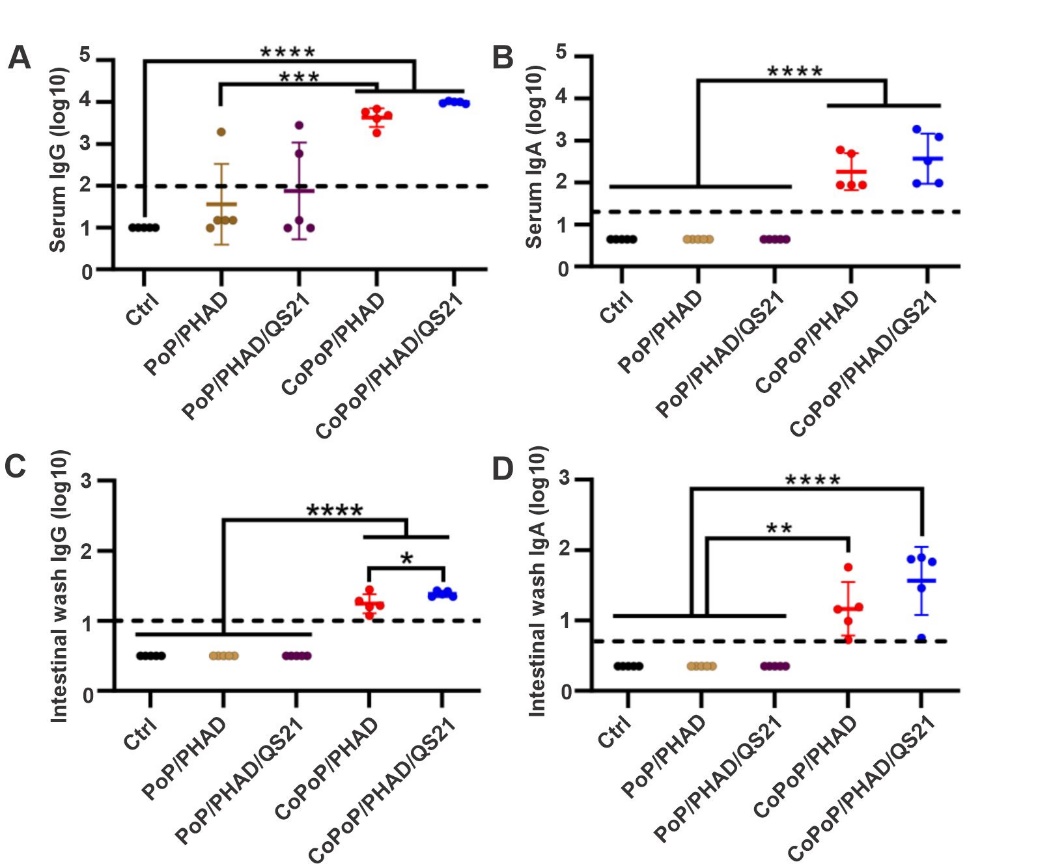


**Figure S3. CfaEad-specific IgG and IgA titer levels induced by different liposome formulations.** ICR mice were immunized with indicated CfaEad vaccines on day 0 and 21. Serum samples and intestinal wash samples were collected on day 42. The antigen dose was 5 µg, the PoP or CoPoP dose 20 µg and the PHAD and QS-21 dose was 8 µg. **A)** Serum IgG titer; **B)** Serum IgA titer; **C)** Intestinal wash IgG titer; **D)** Intestinal wash IgA titer. Mean and standard deviation are indicated. Statistical analysis was performed with Log_10_-transformed values (n=5 mice/group) with statistical comparisons performed by one-way ANOVA followed by Tukey's multiple comparisons test, *p<0.05, **p<0.01, ***p<0.001, and ****p<0.0001.
